# High-throughput mutagenesis using a two-fragment PCR approach

**DOI:** 10.1038/s41598-017-07010-4

**Published:** 2017-07-28

**Authors:** Franziska M. Heydenreich, Tamara Miljuš, Rolf Jaussi, Roger Benoit, Dalibor Milić, Dmitry B. Veprintsev

**Affiliations:** 1 Laboratory of Biomolecular Research, Paul Scherrer Institute, 5232 Villigen PSI, Switzerland; 2 Department of Biology, ETH Zürich, 8093 Zürich, Switzerland; 3 Present Address: Institute of Biochemical Plant Physiology, Heinrich Heine University Düsseldorf, 40225 Düsseldorf, Germany

## Abstract

Site-directed scanning mutagenesis is a powerful protein engineering technique which allows studies of protein functionality at single amino acid resolution and design of stabilized proteins for structural and biophysical work. However, creating libraries of hundreds of mutants remains a challenging, expensive and time-consuming process. The efficiency of the mutagenesis step is the key for fast and economical generation of such libraries. PCR artefacts such as misannealing and tandem primer repeats are often observed in mutagenesis cloning and reduce the efficiency of mutagenesis. Here we present a high-throughput mutagenesis pipeline based on established methods that significantly reduces PCR artefacts. We combined a two-fragment PCR approach, in which mutagenesis primers are used in two separate PCR reactions, with an *in vitro* assembly of resulting fragments. We show that this approach, despite being more laborious, is a very efficient pipeline for the creation of large libraries of mutants.

## Introduction

Scanning mutagenesis, the replacement of selected single amino acid residues by alanine or any other amino acid^1, 2^ is a valuable tool for systematically probing protein functionality. The technique permits mapping of protein–protein interaction interfaces, ligand binding sites and residues important for enzymatic activity or general functionality. Furthermore, it allows protein engineering by combination of mutations with desired characteristics. While mutagenesis was initially limited to very few residues, improvement of mutagenesis techniques now enables scanning of an entire protein sequence of several hundred amino acids. This was demonstrated for both soluble and membrane proteins, including several G protein-coupled receptors (GPCRs) with the aim of stabilising the inherently unstable proteins, reviewed in ref. 3, and for functional mapping of Gα_i1_ and arrestin-1 residues involved in GPCR signalling^4, 5^. As an alternative to using scanning mutagenesis based on predefined sets of mutations, random mutagenesis is also a very powerful approach applied to protein engineering and establishing structure-activity relationships^6–10^.

Generally, alanine-scanning mutagenesis is based on PCR amplification of the recombinant DNA followed by an enzymatic digest of the methylated template DNA using endonuclease DpnI. In the improved version (Liu and Naismith^11^) of the widely used QuikChange™ method (Braman, Papworth *et al*.^17^), both partially overlapping PCR primers contain the mutation which is then inserted in the newly synthesized DNA. The PCR-linearized plasmid is then transformed and repaired (circularized) by homologous recombination in the bacterial cells. Although being very simple, this approach sometimes results in artefacts in the final PCR products^11–13^. Several versions of this method are available for producing site-directed mutants, with varying degrees of complexity and efficiency. All of them could be used for library generation. However, when making a large number (several hundreds) of site-directed mutants, and aiming for 100% coverage of the gene of interest, the differences in efficiency become very important for the overall speed, success rate and cost of library generation.

Here we present a high-throughput mutagenesis pipeline which allowed us to systematically generate alanine scanning libraries of 400 single-point mutations in 6 weeks, with a complete coverage of the protein sequence.

## Results and Discussion

In our pipeline, we combined a two-fragment PCR approach, in which mutagenesis primers are used in two separate PCR reactions, with an *in vitro* assembly of the resulting fragments.

**Figure 1:**
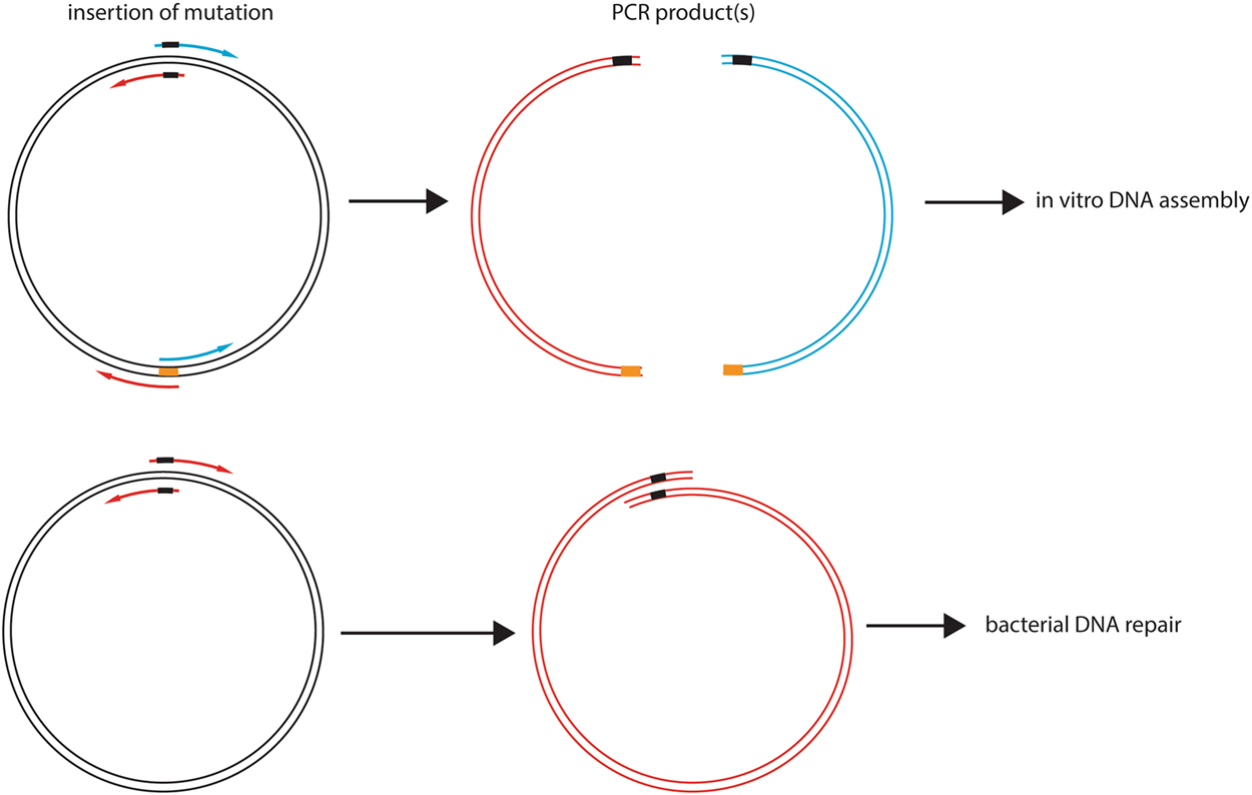
Comparison of mutagenesis techniques. In the two-fragment approach, the two mutagenesis primers are separated into two PCR reactions and combined with one reverse primer on the opposite side of the vector respectively, while the one-fragment approach relies on two mutagenesis primers in one PCR reaction.

The mutagenesis primers are combined with another pair of primers, annealing approximately on the opposite side of the plasmid (Fig. 1). The two vector fragments generated in separate PCRs are combined in the simple and efficient Gibson assembly^14^, merits of which were extensively discussed by Benoit *et al*.^15^. Because shorter fragments are amplified, the PCR is more robust and efficient. This is clearly an advantage over other techniques in which a full-length plasmid is amplified^11, 12, 16, 17^, including the one we have used earlier for scanning mutagenesis of the human G_ai1_ (354 residues, UniProtKB: P63096) and bovine arrestin-1 (404 residues, P08168) proteins^13^. After the initial success of the two-fragment approach in generating several “difficult-to-make” arrestin-1 mutants, we decided to use this strategy for high-throughput scanning mutagenesis of two human GPCRs – cannabinoid CB2 receptor (CB2; 360 residues, P34972) and vasopressin V2 receptor (V2R; 371 residues, P30518).

Here, each vector fragment was amplified by PCR using one mutagenic primer and the appropriate ColE1 primer annealing to the complementary DNA strand within the vector’s bacterial origin of replication (Fig. 1). Although one could design a non-mutagenic primer that anneals anywhere on the vector further away from the mutation site, the origin of replication offers several advantages: (1) many different plasmids share the same origin of replication, so the same pair of the reverse-complementary ColE1 primers can be used in all such cases, (2) only plasmid DNA with a properly assembled replication origin can be replicated in bacterial cells and produce colonies on plates with selection antibiotic and (3) an origin of replication is usually situated on a plasmid approximately “opposite” the gene of interest, which results in more or less balanced sizes of the two vector fragments that are to be assembled.

**Figure 2:**
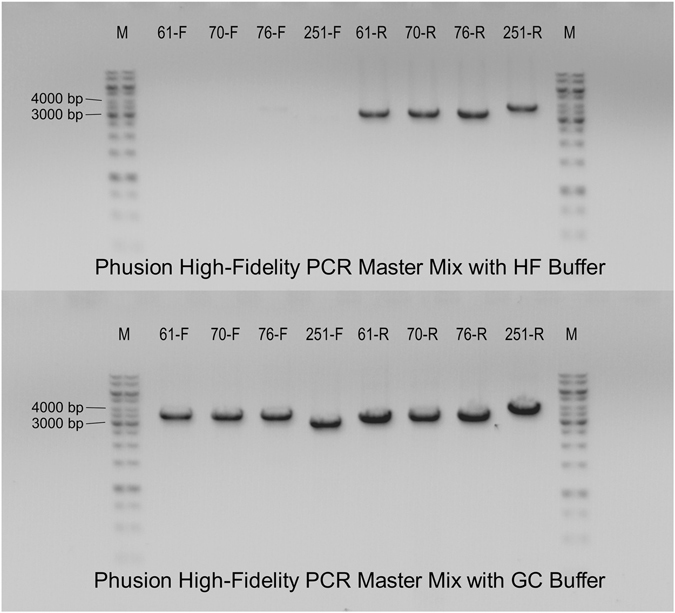
Agarose gel electrophoresis analysis of PCR fragments multiplied by Phusion High-Fidelity PCR Master Mix with H﻿F ﻿B﻿uff﻿﻿e﻿r and Phusion High-Fidelity PCR Master Mix with GC Buffer. Analysed PCRs are labelled as the mutated residue and the letter indicating whether the fragment is multiplied by mutation-specific forward (F) or reverse (R) primer. Expected PCR fragments are between 3000 and 4000 bp long. While using the PCR master mix with GC buffer gave expected fragments in all shown cases, using the Phusion High-Fidelity polymerase with HF buffer failed to multiply four fragments.

The mutagenic primers were designed in a high-throughput way using the program AAscan^13^. The amino acids of the target protein were changed to alanine, while alanines were exchanged for glycines (V2R) or valines (CB2). The alanine and glycine substitutions remove the side chain of the amino acid and are used for probing its role in protein function. Valines are one of the prevalent types of amino acids in membrane proteins and promote stability of transmembrane helices. These substitutions are generally well tolerated and used for thermostabilisation of GPCRs ﻿(reviewed in refs 3, 18 and 19). For both receptors, we have used the mammalian expression vector pcDNA4/TO. In preliminary experiments, we tested different DNA polymerases and optimized PCR conditions for the preparation of 30 randomly chosen CB2 mutants, analysing the PCR products on agarose gels (Fig. 2). Success of mutagenesis was dependent on DNA polymerase and PCR conditions (see Materials and Methods). The same PCR conditions used for CB2 were also successfully applied in high-throughput mutagenesis of V2R without any further optimization. The PCR elongation time was calculated based on the longest fragment to be made in the whole alanine scan. We used a step-down PCR protocol^20^ with a decrease of annealing temperature of 0.5 °C per cycle to accommodate the variation of primer melting temperatures inherent to a large high-throughput screen.

**Figure 3:**
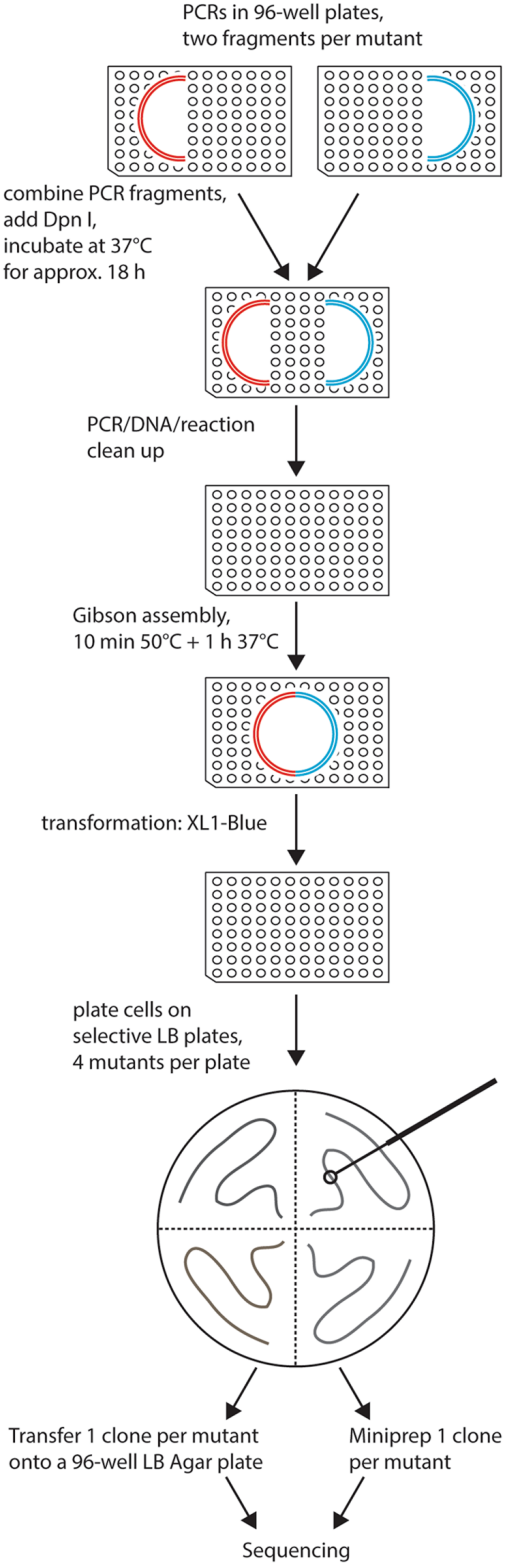
Overview of the mutagenesis technique. Two PCR reactions are done per mutant, in each of them approximately half of the vector is amplified. Two fragments containing one mutation are combined, followed by DpnI digestion at 37 °C overnight. Reaction clean-up is performed to purify DNA fragments, which are then assembled by Gibson assembly reaction. Bacteria are transformed with the resulting circular plasmid and plated on selective LB agar plates. One clone per mutant is sent for sequencing either on a selective LB agar 96-well plate or as purified DNA. All steps excluding the plating of the bacteria are done in 96-well plates.

**Figure 4:**
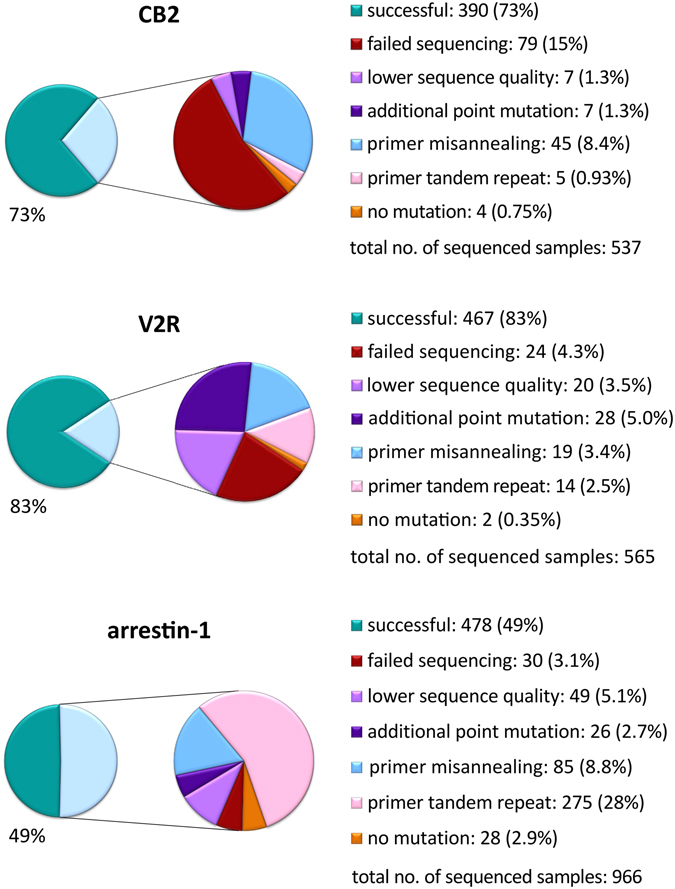
Results of alanine scanning mutagenesis on two G protein-coupled receptors, cannabinoid CB2 receptor (CB2) and vasopressin V2 receptor (V2R) compared with the one-fragment mutagenesis approach used for arrestin-1 (Sun, Ostermaier *et al*.^13^). Reason for failure and corresponding percentage within the total number of DNA samples sent for sequencing are given. In case of V2R and arrestin-1, the total number of analyzed samples is smaller than the sum of individual categories as in a few instances several failure reasons could be found within a sequenced sample. Category “failed sequencing” relates to instances with very noisy peaks or no peaks at all in a sequencing electrophoretogram. On the other hand, “lower sequence quality” denotes interpretable sequence traces that had some artefacts or were too short to provide reliable information about success of mutagenesis.

After PCR, the two matching fragments carrying one mutation were combined without checking PCR products on an agarose gel (Fig. 3). The methylated template DNA in a mixture of fragments was digested with DpnI at 37 °C for 18 h. This resulted in a very low background of wild-type template plasmid (Fig. 4). DpnI digestion was followed by DNA clean-up in order to remove remaining primers, enzymes and deoxynucleotides, crucial for the success of the following enzymatic reaction. A Gibson assembly at 50 °C for 10 min followed by 1 h at 37 °C with 1 μL cleaned-up DNA and 3 μL 1.33× Gibson assembly mix (see Materials and Methods) proved to be sufficient for the high-throughput applications. The ratio of the fragments used in Gibson assembly was not adjusted for the high-throughput mutagenesis. Instead, both fragments were combined directly after the PCR reaction to save time and reagents. A separate clean-up of fragments and adjustment of fragment ratios might increase success rates in low-throughput applications. Transformations were done with 2 μL assembly product and 20 μL chemically competent *Escherichia coli* XL1-Blue cells. As shown for V2R mutants, transformed cells could be plated on a quarter of selective lysogeny broth (LB) agar plates to save space without losing efficiency of mutagenesis. Alternatively, bacteria could be plated on 24-well plates using an expanding pipette^21^. It should be noted that 68 CB2 transformants gave no visible colonies on the selection plates. We hypothesized that the corresponding PCRs were not successful, so we repeated them using another DNA polymerase and the corresponding PCR conditions (see Materials and Methods).

Colonies were sent for sequencing on 96-well LB agar plates with the appropriate selection antibiotic. Initially, we sent out only one colony per mutant to reduce sequencing costs. If the first sequencing reaction was not successful, we sequenced DNA from one to three additional colonies. Difficult CB2 mutants (14, 4%) were obtained by repeating PCR, analysing products on agarose gels and cutting the right bands out in case of significant side-products. Alternatively, the rest of the missing V2R mutants (3, 0.8%) were obtained by repeating the PCR with two mutagenic primers in the same reaction mixture (Fig. 1), followed by *in vivo* recombination in the *E. coli* Mach1 strain^22^, according to our earlier one-fragment approach^5, 13^. When few mutants remain to be cloned, we tend to try different approaches in parallel and/or send more clones at once. Therefore, the standard single-fragment approach was used alongside the two-fragment method.

**Figure 5:**
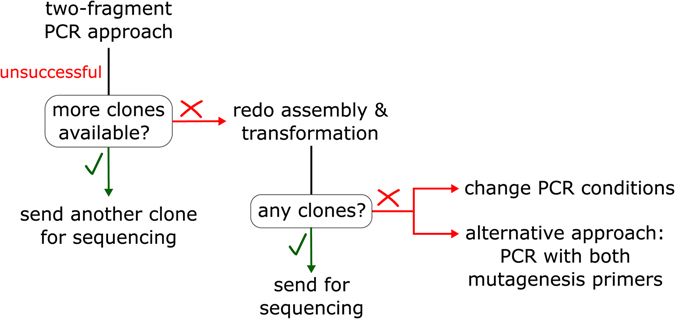
Troubleshooting scheme. For mutants which were not obtained in the first round of cloning, another clone can be sequenced, if it exists. Furthermore, the Gibson assembly reaction can be redone with the same purified DNA fragments, followed by bacterial transformation and sequencing. For missing mutants, PCR conditions can be changed or PCR containing both mutagenesis primers (one-fragment approach) can be applied.

Once PCR conditions have been optimized for the particular vector used, the mutagenesis is straightforward. Most steps are done in 96-well plates, contributing to the protocol’s simplicity. Most mutations not obtained in the first round of mutagenesis can be obtained by sending additional clones for sequencing, while others can be obtained by repeating Gibson assembly or PCR, depending on the cause of failures (see Fig. 5).

We defined cloning efficiency as a percentage of the expected sequences obtained in all successful sequencing reactions (excluding sequencing failures and non-informative sequencing data). We use this term when discussing occurrence of primer tandem repeats, which were the main cause of failure in the one-fragment approach used in arrestin-1 high-throughput mutagenesis we use as a comparison. However, it should be noted that sequencing failures could also be caused by unsuccessful cloning, e.g. when a part of plasmid to which sequencing primer should anneal is missing. Because we cannot distinguish a true sequencing failure from a cloning failure in our data, we decided to use an overall success rate of the method as well. It takes into account all encountered practical difficulties and it is defined as a percentage of the expected sequences obtained in all sequencing attempts (including failed sequencing and non-informative sequencing data). In our opinion, this information is valuable in the context of high-throughput site-directed mutagenesis with direct repercussions on expected costs.

Taken together, we successfully generated complete alanine scanning libraries of the two GPCRs. For V2R, 467 out of 565 sequencing reactions confirmed the designed single amino acid mutations, which correspond to an overall success rate of 83%. For CB2, we obtained the right sequences for 390 out of 537 colonies sent for sequencing (73%). The lower efficiency in this case was mostly due to failed sequencing or lower quality of sequencing data not allowing their reliable interpretation as well as primer misannealing resulting in large deletions (Fig. 4). In general, the most common problems encountered in these projects were unsuccessful sequencing attempts, too short sequence reads and deletions of a part of the vector. We observed very few cases of vectors bearing no mutation (unsuccessful DpnI digestion reaction) or an additional mutation (PCR error or mutation in a primer oligonucleotide).

We also used the same two-fragment cloning approach to prepare twenty N- and/or C-terminally truncated constructs of *Arabidopsis thaliana* and tomato ethylene receptor 1 (ETR1) – two plant membrane proteins with an extensive cytoplasmic domain. Out of 50 successfully sequenced DNA samples, 45 had the correct sequence. Although the remaining 5 had the correct truncation, they also contained an additional point mutation, presumably introduced as a DNA polymerase error in PCR. While the Gibson method is an established cloning method, these results suggest that it is very well suited for general high-throughput applications.

**Figure 6:**
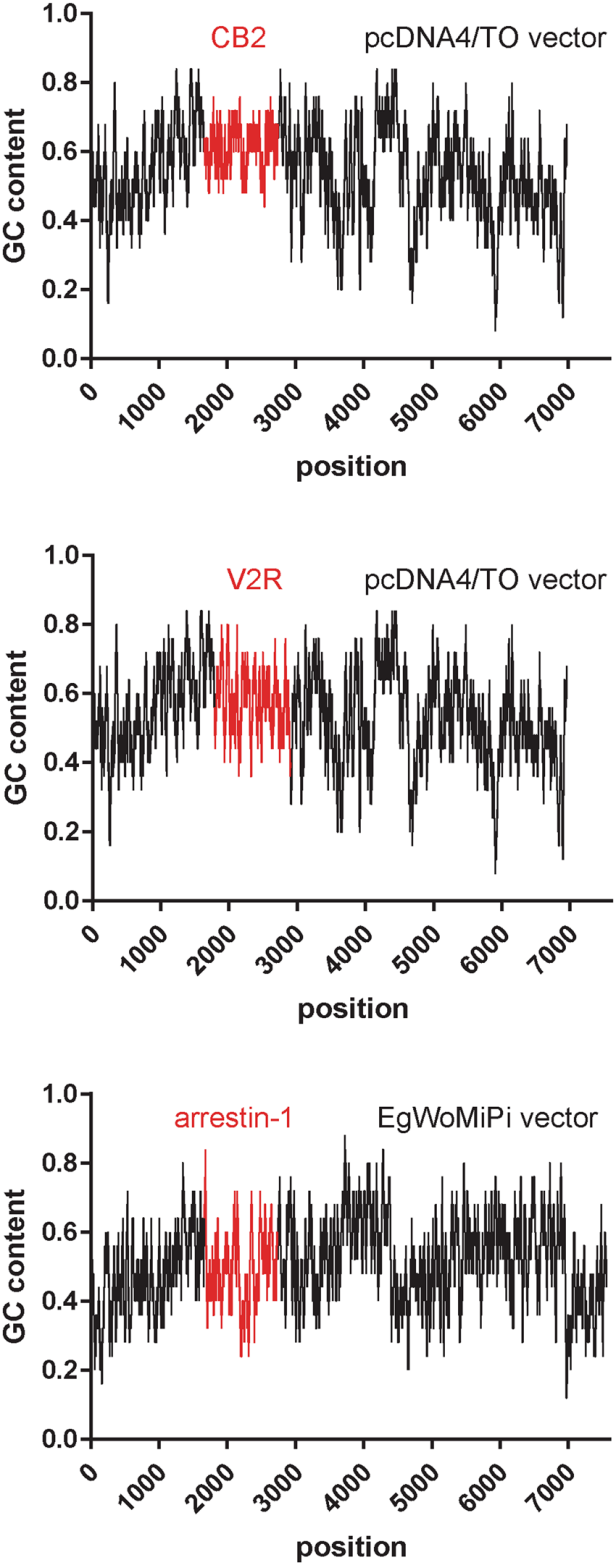
GC content of the plasmids used for the generation of the mutagenesis libraries. For each nucleotide position in the plasmid sequence, GC content was calculated using a 25 bp sliding window. The mutated sequences are highlighted in red.

The success of mutagenesis for a given template would, naturally, depend on a number of parameters in addition to the mutagenesis method chosen. For example, the length of the plasmid to be amplified needs to be considered, as well as extremely high or low GC content^13^. In our case, the pCDNA4/TO vector encoding for fusions of the CB2 and V2R constructs used in this work have the same length of 7 kbp and an average GC content of 55 and 54%, respectively (Fig. 6). The length of the plasmid encoding for arrestin-1 fusion to mCherry^4^ is 7.6 kbp and it is also similar in GC content (50.4%). The constructs encoding for the ETR1 were in the range of 6.2–7.5 kbp. Based on the two parameters that can dramatically affect the PCR efficiency, the length and GC content, these samples are relatively similar. The plasmid size is very critical for co-transformation cloning, where overlapping fragments are simply used to co-transform *E. coli*, without *in-vitro* treatment with In-Fusion or assembly mix^15^. However, the assembly mix is efficient at combining DNA fragments up to several hundred kilobases, a size that is never reached with plasmids^14^. If insert-plasmid mixtures are combined *in vitro* using assembly mix, as in our method, the limiting factor is the PCR. Because we use two fragments and 6 kb can be robustly amplified today, the method works for plasmids up to at least 12 kb. The larger plasmid size also has a slight negative effect on transformation efficiency, but this is not a problem with competent cells of reasonably high transformation efficiency. In comparison to the method previously used in our lab with both mutagenic primers in one PCR and recombination in Mach1 cells^13^, we found that a number of instances with tandem repeats of the primer sequence before or after the mutation site was drastically reduced (Fig. 4), resulting in increased cloning efficiency (86% and 90% compared to 54% of successfully sequenced samples). The main improvements clearly came from performing two separate PCR reactions which amplify two fragments of the target vector.

In our opinion, even higher mutagenesis success rates can be achieved using a novel, not expensive and high-fidelity DNA polymerase (such as Q5), longer elongation times (at least 30 s/kbp) and less PCR cycles in combination with annealing temperatures not lower than 60 °C (for the primers designed as described in Materials and Methods). Furthermore, the Hot Fusion method^15, 23^ can be used for assembling two fragments instead of the Gibson reaction, which further reduces the cost of mutagenesis by omitting a DNA ligase.

The two-fragment approach presented here can be further extended to a three-fragment approach where the whole plasmid backbone is prepared by restriction digestion, mutagenesis is then done with mutagenesis primers and primers flanking the gene of interest, followed by PCR clean-up and Gibson assembly. This could be a very efficient way of making mutants, especially in the GC rich regions that are challenging for the PCR. An additional benefit is that the backbone, since it is only produced once, can be verified for the absence of mutations and deletions, something that is not practical with the two-fragment approach. It needs to be noted that, from our experience, it is more difficult to assemble three fragments successfully, especially when the molar ratio of the assembling fragments has not been chosen carefully. In our two-fragment high-throughput approach, we do not measure or adjust fragment concentration before the assembly reactions.

Although there are many general cloning methods developed and kits commercially available, it is of crucial importance to have a highly-efficient, simple, fast and cost-effective method when it comes to generating libraries comprised of hundreds of mutants with 100% coverage. Even though the presented high-throughput mutagenesis and cloning pipeline is slightly more laborious than other established mutagenesis protocols, we found this to be a convenient and efficient alternative.

## Materials and Methods

### Software for primer design and sequence analysis

In this work, we used software specially developed for primer design and sequence analysis in high-throughput scanning mutagenesis^13^. The AAscan, PCRdesign and MutantChecker are an open source software written in an open source multi-platform Pascal-Lazarus environment^24, 25^ and are freely available from the authors’ web site (https://www.psi.ch/lbr/aascan).

### Primer design

For human CB2 and V2R genes in pcDNA4/TO vector (both plasmid sizes 7.0 kb) we designed the mutagenic primers in a high-throughput way by using the program AAscan and the following parameters: a primer length 18–60 nt (nucleotides), a minimum GC clamp of 2, a melting temperature (*T*_m_) of 60–70 °C with a maximum Δ*T*_m_ of 5 °C for a pair of primers. Our designed primers, excluding the mismatching bases, had annealing temperatures in a range 60–68 °C. The minimal overlap of the two mutagenic primers was 21 nucleotides for CB2 and 15 for V2R. A minimum of 13–15 bases is needed for both Gibson assembly and bacterial recombination to work^26, 27^. A too long overlap may restrict primer design options of the program, which may lead to a decrease in mutagenesis success rates. In several cases of CB2 mutagenesis, the value of 21 nucleotides had to be decreased in order to design primers within the given restraints. Longer overlap of the primers used for CB2 mutagenesis may be an explanation for the lower success rate compared to V2R mutagenesis. Codons were chosen according to their frequency in the expression host and according to the GC content of the gene. Very high and very low GC contents should be avoided. For mammalian expression vectors, we chose the codons GCC/GCT for alanine, GGC/GGA for glycine and GTG/GTC for valine. For genes with high GC contents it could be advisable to choose GCT/GCA for alanine, GGA/GGT for glycine and GTT/GTA for valine, provided that the host organism is able to translate these codons efficiently. Due to consecutive cytosine bases in the CB2 gene sequence, several primers designed in AAscan had to be manually modified by introducing other degenerate codons in order to avoid G-repeats in the reverse (antisense) primers. G-repeats are prone to formation of G-quadruplex secondary structure (Burge, Parkinson *et al*. 2006) and might thus interfere not only with the oligonucleotide production and quantification, but might also reduce PCR efficiency. Primers used for the C- and/or N-terminal truncations of ethylene receptor 1 from *Arabidopsis thaliana* (AtETR1, UniProtKB: P49333; original DNA sequence) and tomato (*Solanum lycopersicum*; LeETR1, Q41342; DNA sequence codon-optimized for *E. coli* expression) in pET16b vectors were designed in program PCRdesign^13^ with the following parameters: a primer length 16–50 nt, an overlap 21 bp (base pairs), a minimum GC clamp of 2, optimized GC content and a minimum *T*_m_ of 60 °C.

The sequences of the ColE1 origin primers used in two-fragment cloning were GGAGCGAACGACCTACACCGAACTGAGATACCTACAGCG and CGCTGTAGGTATCTCAGTTCGGTGTAGGTCGTTCGCTCC. All primers were ordered as desalted oligonucleotides and used without further purification. The primers for high-throughput scanning mutagenesis were synthesized by Integrated DNA Technologies and delivered in 96-well plates at a concentration of 100 or 150 μM in 10 mM Tris-HCl, 0.1 mM EDTA (ethylenediaminetetraacetatic acid), pH 7.5. Other primers were ordered from Microsynth or Sigma-Aldrich in lyophilized form.

### PCR set-up and high-throughput mutagenesis

PCR conditions were first tested for a random set of 30 CB2 mutants using Phusion High-Fidelity PCR Master Mix with HF ﻿Buffer, Phusion High-Fidelity PCR Master Mix with GC Buffer (New England Biolabs, NEB) and KOD Hot Start Master Mix (Merck Millipore). Based on analysis of the PCR products on agarose gels (Fig. 2), Phusion High-Fidelity DNA Polymerase with GC buffer was chosen for high-throughput mutagenesis, as in most instances, it gave the expected DNA bands with no or very little non-specific products. Interestingly, we noted that the absence of a PCR fragment band on an agarose gel does not necessarily mean that the mutagenesis will be unsuccessful. In one specific case (I205A in CB2) we obtained more that 50 colonies when plated on a whole LB agar plate as described below. Two out of three sequenced clones gave the expected mutant. Therefore, we do not think that detecting the correct PCR product on the agarose gels with ethidium bromide staining is a prerequisite to have successful mutagenesis. Even if PCR multiplication apparently fails, a small, undetectable amount of a PCR fragment coming from primer extension using the original template can lead to successful mutagenesis using the described method^15, 28^. It is also possible that the PCR reaction was not efficient enough to be detected by ethidium bromide staining.

For the two-fragment approach, each mutagenesis primer was combined with the matching ColE1 primer, giving two PCR products per mutation. For the one-fragment approach, the two mutagenesis primers were combined in one PCR reaction, which required a longer elongation time compared to the two-fragment approach. PCRs were carried out in 20 μL final volume with 1 ng template DNA, 300 nM of each primer and, in case of CB2 only, 3% (v/v) DMSO (dimethyl sulfoxide). Good results were obtained with the following thermocycling setup using Phusion High-Fidelity DNA Polymerase with GC buffer: initial denaturation at 98 °C for 1 min, followed by 20 step-down thermal cycles, each comprising denaturation at 98 °C for 20 s, annealing from 65 °C down to 55.5 °C for 30 s (0.5 °C decrement per cycle), and elongation at 72 °C for at least 25 s per kbp of the expected PCR product (in total up to 2 min), then 10 thermal cycles with the constant annealing temperature (denaturation at 98 °C for 20 s, annealing at 62.5 °C for 30 s, elongation at 72 °C with the same time period as used in the step-down cycles), and final elongation at 72 °C for 3 min completed with a hold at 10 °C. When using KOD Hot Start Master Mix, the following thermocycling setup was used: Initial denaturation at 95 °C for 2 min, followed by 20 step-down thermal cycles, each comprising denaturation at 95 °C for 20 s, annealing from 65 °C down to 55.5 °C for 10 s (0.5 °C decrement per cycle), and elongation at 70 °C for at the appropriate time (up to 135 s), then 5 thermal cycles with the constant annealing temperature (denaturation at 95 °C for 20 s, annealing at 62.5 °C for 10 s, elongation at 70 °C with the same time period as used in the step-down cycles), completed with a hold at 10 °C. For truncations of the AtETR1 and LeETR1 plasmids by the two-fragment approach, a slightly different PCR protocol was used: 20 μL PCR mixture prepared with nuclease-free water (Cell Signaling Technology) contained 1 ng DNA template, 500 nM each primer, 200 μM each dNTP (deoxynucleoside triphosphate; NEB), 0.4 U μL^−1^ Phusion High-Fidelity DNA Polymerase (NEB), and either 1× Phusion buffer HF (NEB) or 3% (v/v) DMSO (NEB) with 1× Phusion buffer GC (NEB). Also, PCR thermocycling described above was modified in case of the ETR1 plasmids to have: (1) 11 step-down cycles with annealing starting from 65 °C down to 60 °C (i.e. decreasing by 0.5 °C in each subsequent cycle); (2) elongation time of at least 30 s per kbp of the target product (up to 200 s for the longest PCR products); and (3) final elongation time of 5 min.

### DNA template digestion and reaction clean-up

After PCR, the two fragments carrying one mutation in a GPCR gene were combined already before digestion and purification. To reduce background, methylated template DNA was digested by adding 0.5 μL DpnI enzyme (20 units μL^−1^ from NEB or 10 units μL^−1^ from Thermo Scientific) to a final PCR mixture. The mixtures with DpnI were incubated at 37 °C for 18 h (GPCR mutagenesis) or 2 h (ETR1 truncated products), however the digestion time can be shortened if necessary, provided the template amount in PCR and DpnI amount are optimised beforehand. The reaction was cleaned-up using a 96-well ZR-96 DNA Clean-up Kit (Zymo Research), MinElute Reaction Cleanup Kit (Qiagen) or Illustra GFX PCR DNA and Gel Band Purification Kit (GE Healthcare) according to the manufacturer’s instructions. We used a low elution volume of 10 μL to obtain higher DNA concentrations and, in case of two-fragment cloning, a more efficient Gibson assembly.

### Gibson assembly

Cleaned PCR products with two fragments were assembled *in vitro* using Gibson assembly^14^. Twelve mL 1.33× Gibson assembly mix was prepared in-house using 6.4 μL 10 U μL^−1^ T5 exonuclease (NEB), 200 μL 2 U μL^−1^ Phusion High-Fidelity DNA polymerase (NEB) and 1.6 mL 40 U μL^−1^ Taq DNA ligase (NEB) in isothermal reaction (IT) buffer (5% (w/v) PEG-8000 (poly(ethylene glycol) 8000), 100 mM Tris-HCl pH 7.5, 10 mM MgCl_2_, 10 mM DTT (dithiothreitol), 1 mM β-NAD (β-nicotinamide adenine dinucleotide), 200 μM of each dNTP). IT buffer was prepared as a 5× stock in nuclease-free water. In case of the CB2 and V2R mutants, 1 μL cleaned-up DNA mixture was added to 3 μL 1.33× Gibson assembly mix and incubated at 50 °C for 10 min followed by 1 h at 37 °C. In case of CB2, with 21 bp overlap within a pair of mutagenic primers, we observed more colonies on selective LB agar plates when Gibson reaction was performed for 1 h at 50 °C. For ETR1 truncations, 2.5 μL 1.33× Gibson assembly mix was combined with 1 μL of the longer PCR product and 1.5 μL of the shorter one, without determining DNA concentration of each cleaned-up PCR product, and the assembly was conducted at 50 °C for 1 h.

### Cell transformation

Chemically competent *Escherichia coli* XL-1 Blue cells were prepared by the Inoue method^29, 30^ and had a transformation efficiency of at least 10^7^ colonies per 1 µg plasmid pBR322. The assembled fragments were transformed into the competent cells using 20 μL of cell suspension and 2 μL assembly mix for GPCR mutagenesis or 50 μL cell suspension and 5 μL assembly mix for ETR1 mutagenesis. Cells for transformations were thawed on ice, aliquoted, incubated with DNA for 10–20 min, heat shocked for 1 min at 42 °C, incubated on ice for 2 min and plated on LB agar plates with 150 μg mL^−1^ ampicillin. A quarter of LB agar plate was used for each V2R mutant, while the transformed cells of other constructs were spread over a whole plate. Plated cells were incubated at 37 °C for 16–24 h.

PCR reactions, heat shock for transformation and Gibson assemblies were done in 96-well PCR plates (Eppendorf) using Mastercycler pro S or Mastercycler gradient thermocyclers (Eppendorf).

### Plasmid DNA preparation and sequencing

In the high-throughput CB2 and V2R mutagenesis, one cell colony per mutation was transferred into a well of a 96-well LB agar plate with 150 μg mL^−1^ ampicillin and sent for plasmid preparation and sequencing by the GATC Biotech Company. Sequencing results were analyzed with the program MutantChecker^13^. In case the mutant was not obtained in the first round, another clone was sent for sequencing. If there were no more clones, PCR with modified conditions and/or Gibson assembly was repeated. ETR1 plasmids were prepared using NucleoSpin Plasmid Kit (Macherey-Nagel) and sequenced by the Biological-Medical Research Centre (BMFZ) at the Heinrich Heine University Düsseldorf.

### Troubleshooting/Later rounds of mutagenesis

A small number of mutants have proven difficult to make, 14 (4%) in case of CB2 and 3 (0.8%) in case of V2R. In the CB2 mutagenesis, a large deletion in the plasmid was a most common problem. This is a clear indication of a primer misannealing in PCR, probably related to a higher GC content (61%) in the CB2 gene, while no correlation with high GC content in the primer itself was observed (Supplementary Data). To obtain missing mutants, PCR was repeated using different DNA polymerase (Hot Start Master Mix). In the third round of mutagenesis, PCR was repeated using both Phusion High-Fidelity DNA Polymerase with GC buffer and KOD Hot Start Master Mix with the conditions as described above, but longer elongation times were used (135 s). PCR fragments were analysed on agarose gels (Fig. 2) and conditions with stronger bands and less side-products were chosen. Several specific PCR products were purified on an agarose gel before applying Gibson assembly (QIAquick Gel Extraction Kit Protocol was used according to the manufacturer’s instructions). Gibson assembly was done by mixing 2.5 μL cleaned-up DNA and 7.5 μL 1.33× Gibson assembly mix, followed by incubation at 50 °C for 1 h. Since few colonies grew on selective plates, Gibson assembly was repeated by incubation at 50 °C for 10 min and 37 °C for 1 h. With these samples, there was no indication that one incubation protocol is better than the other. Several colonies per mutant were sequenced. One mutant was obtained by preparation of DNA from ten colonies, following by selection of the full-sized plasmid by plasmid linearization by restriction endonuclease NdeI (NEB) and analysis on an agarose gel. All of seven non-truncated samples had the appropriate mutation. The last missing mutant always gave little or no colonies on selective LB agar plates, therefore this mutant was ordered from GenScript due to time limitations.

An alternative approach was used for obtaining the three remaining V2R mutants. In this case, a PCR was repeated, but with the two mutagenic primers in the PCR mixture (“single-fragment” approach) and a longer extension time which is sufficient for full plasmid amplification (Sun, Ostermaier *et al*.^13^). Linear PCR products were digested with DpnI and directly transformed into the chemically competent *E. coli* Mach1 strain for *in vivo* recombination, in which case SOC medium was added after transformation followed by 2 h recovery at 37 °C to allow bacterial DNA repair before plating on LB agar plates with the selective antibiotic.

### Data availability statement

The results of data analysis are included in the supplementary information.

## Electronic supplementary material

Supplementary Dataset 1
